# Dietary Habits before and during the COVID-19 Epidemic in Selected European Countries

**DOI:** 10.3390/nu13051690

**Published:** 2021-05-16

**Authors:** Magdalena Skotnicka, Kaja Karwowska, Filip Kłobukowski, Eliza Wasilewska, Sylwia Małgorzewicz

**Affiliations:** 1 Department of Commodity Science, Medical University of Gdańsk, 80-211 Gdańsk, Poland; skotnicka@gumed.edu.pl (M.S.); kaja.karwowska@gumed.edu.pl (K.K.); filip.klobukowski@gumed.edu.pl (F.K.); 2 Department of Allergology, Medical University of Gdańsk, 80-211 Gdańsk, Poland; ewasilewska@gumed.edu.pl; 3 Department of Clinical Nutrition and Dietetics, Medical University of Gdańsk, 80-211 Gdańsk, Poland

**Keywords:** dietary habits, COVID-19 pandemic, obesity, physical activity

## Abstract

During the so-called “second wave of the pandemic” in Europe, the authors conducted a cross-sectional online survey that aimed to examine changes in dietary habits and associated practices, as well as physical activity during the COVID-19 pandemic and before the onset of lockdowns in three European countries: Poland, Austria and the United Kingdom. Methods: The online observational study, both prospective and retrospective, conducted with the use of social media for the distribution of an anonymous online questionnaire, was completed from 1 October to 30 October 2020, during the second wave of the pandemic in Europe. The study encompassed a total of 1071 adults from Poland (*n* = 407), Austria (*n* = 353) and the United Kingdom (*n* = 311). Results: The results of this study indicate that the COVID-19 confinement period influenced eating behavior and the level of physical activity in a group of adult residents of Poland, Austria and the United Kingdom. The general shopping frequency decreased, regardless of the place and manner. However, there was an increased interest in online grocery shopping. The resulting data revealed an increased frequency of the daily consumption of food products such as dairy, grains, fats, vegetables and sweets (*p* < 0.05). A rise in the frequency of purchasing frozen goods and food with long shelf life has also been observed. The changed workplace and working conditions or unemployment probably affected a perceptible rise in alcohol consumption (*p* = 0.02). In turn, physical activity levels markedly decreased, which reflected the body mass changes. Conclusion: The dietary habits in the studied countries have changed as a result of the pandemic situation. They contribute to the aggravation of the problem of excess body weight and its health consequences.

## 1. Introduction

The detection of a new disease (COVID-19) caused by the Sars-Cov-2 virus in Hubei Province, China, in December 2019 marked the commencement of global problems in the areas of labor, economy, production and health. The situation soon proved grave enough to warrant the declaration of a pandemic in March 2020 [1,2]. In the face of this phenomenon, all countries, each at its own pace, made restrictive decisions about the control, hygiene and regulations governing social life in the new reality. To slow down the spread of the virus, most countries opted for radical measures that placed restrictions on the daily lives of millions of people across the world. Primary constraints concerned the maintenance of proper hygiene, social distancing and reduced mobility. Many countries recommended switching to a remote work mode when possible, which entailed prolonged confinement in the same place and promoted a sedentary lifestyle. As the situation drew on, many citizens experienced pandemic-induced stress, which affected their behavior and food choices, while simultaneously limiting their physical activity (PA) [3]. 

Furthermore, the lockdown affected grocery shopping practices, even though the food supply chains operated continuously (albeit with limitations). The COVID-19 pandemic and the obligation of self-isolation and social distancing disrupted ordinary consumer shopping habits. Many authors reported dietary habit changes during the pandemic in several countries [4,5] among people of various age groups [6] with a range of medical conditions [7,8]. Most commonly, the changes involved the entrenchment of adverse eating habits. Yet, in some cases, the pandemic period allowed for rectifying deficient dietary patterns developed earlier in life [9]. 

In time, some restrictions evolved in view of the epidemic situation in individual countries. However, the beginning of September brought about an upsurge of cases in European countries and necessitated the reinstatement of the earlier restrictions or the imposition of new ones. 

For the most part, human behavior is the force of habits determined by cultural, social and economic factors. Habits are forged in the process of repeating the same responses in particular contexts. Dietary choices are made virtually every day, driven by habitual response, and are extremely stable. However, when the components that make up our environment change, our food choice habits may change as well [10]. Hence, the hypothesis that the pandemic period, which patently constitutes an environmental disruption, could provoke dietary habit changes. 

During the so-called “second wave of the pandemic” in Europe, the authors conducted a cross-sectional online survey aimed to examine changes in dietary habits and associated practices as well as physical activity during the COVID-19 pandemic and before the onset of lockdowns in three European countries: Poland, Austria and the United Kingdom. During the pandemic, the governments of those countries sought to limit the spread of the virus by obliging the citizens to cover their mouths and noses while using public areas and transport, as well as to follow a 1.5 m social distancing rule.

During the survey period, the restrictions in Poland partially prohibited the activity of sports clubs and gyms. Only sports club members could use the available infrastructure to prepare for the competitions. Furthermore, the limitations struck restaurant owners who had to limit the number of customers per square meter [11]. The inhabitants of Austria experienced similar restrictions, but preserved access to restaurants, hotels, swimming pools and gyms adhering to social distancing rules [12]. The situation exacerbated only after 3 November 2020, when our survey had been already completed. Starting from 1 October 2020, the British government introduced official social measures to reduce the spread of the virus. Like in Poland, the country was divided into tiers, with the local number of new cases dictating the level of restrictions. In general, fitness clubs and other sports venues could operate, but pubs and bars were closed [13]. 

The research was motivated by numerous reports on the negative impact of pandemic isolation on the eating habits of various population groups and societies. Therefore, the aim of our study was to investigate eating habits before and during COVID-19 and to estimate the differences between the selected countries and to identify adverse changes that may lead to further health consequences. In all likelihood, the consequences of COVID-19 and the associated isolation will affect the model of life and differ from country to country in a manner determined by not only the epidemic situation but also such factors as the level of socio-economic development and the cultural background. A comparison between strikingly different countries will reveal whether the dietary habits have changed at all and highlight the differences between the examined countries in this respect. An analysis of the findings will provide an insight into the level of changes in health-related behavior. In this context, it will provide healthcare institutions with information important for the planning of prevention measures throughout the pandemic situation and upon its resolution. 

## 2. Material and Methods

### 2.1. Participants

The online retrospective observational study, conducted with the use of social media for the distribution of an anonymous online questionnaire, was completed from 1 October to 30 October 2020, during the second wave of the pandemic in Europe. The target sample included adults aged 18–90 from Poland, Austria and the United Kingdom. The choice of these countries was dictated primarily by a similar course of the pandemic and a similar schedule of introduced restrictions. At the same time, the inhabitants of these countries showed a different approach to the imposed restrictions. The selected countries are characterized by similar eating behavior. Attention was also paid to the ethnic diversity and the general economic situation of the analyzed countries and different cultural conditions. The participation was voluntary, and all the participants accepted the patient information form together with the presentation of the study. The first page of the online survey contained a note about informed consent to participation in the study, to which the respondent expressed their agreement. The study conforms to the ethical principles of non-maleficence, beneficence, justice and autonomy enshrined in the ethical resolutions of each country in accordance with the Declaration of Helsinki (2000). Personal data and domain data were anonymous in accordance with the European General Data Protection Regulation (GDPR 679/2016). In view of the anonymous character of the online survey and the impossibility of tracking sensitive personal data, the approval of an ethics committee was not required (NKBBN/487/2021).

### 2.2. Study Design

Data collection for this cross-sectional study involved the use of an anonymous online questionnaire composed of 25 questions about dietary habits before and during the pandemic. Questions 1–5 related to the frequency of shopping and the place where the food products were obtained. Questions 6–9 focused on the method of obtaining meals. Questions 10–24 concerned the frequency of consumption of selected food products. The last question was about the frequency of physical activity (Supplementary Materials Questionnaire S1). The questionnaire included a total of 50 original questions, with 25 questions concerning habits before the pandemic and an identical 25 questions concerning habits during the lockdown. The questions were developed based on the validated FFQ6 questionnaire compiled by Wądołowska et al., Vienna Food Record and Food Frequency Questionnaire provided by the EPIC-Norfolk study [14,15,16]. At the end of the questionnaire, there were questions about socioeconomic status and changes in body weight based on subjective assessment (without quantitative data). The questions concerned the frequency of shopping for and consuming particular groups of food products. The respondents could choose among six answers: a–never or almost never, b–once a month, c–several times a month, d–several times a week, e–every day and f–several times a day. With the exception of the first question about frequency of eating 4–5 meals a day, which had only five possible answers: a–never or almost never, b–once a month, c–several times a month, d–several times a week and e–every day.

The international character of the study prompted the authors to perform questionnaire validation. The validation process consisted of two parts: translation and assessment of the qualities exhibited by the translated tool. The main goal behind validation was to gain the ability to compare findings on the pandemic-related food behavior on an intercultural (international) level. Validation involved cultural adaptation intended at intercultural comparison (the ability to compare findings of the questionnaire on the intercultural level) and the practical application of the questionnaire in the United Kingdom and Austria. To produce a tool that will assess certain characteristics in the United Kingdom and Austria with the highest possible accuracy, that tool must be developed in consideration of the local profile of the target country. Three versions—a Polish version, the translation (to German and English), and the back translation to Polish—were compared in terms of homogeneity, i.e., the criterion of functional equivalence. The assessment concerned the viability of using the questionnaires in German and English for the assessment of the same aims as the Polish version. The accuracy and reliability of the scale were assessed with statistical methods such as Cronbach’s alpha and the correlations between the collected socio-demographic data and the findings obtained in individual areas and the entire test. Internal consistency was assessed with the test of the Cronbach’s alpha coefficient and the discriminatory power of an item. The test indicated the Cronbach’s alpha value at the level of approximately 0.8, which signifies that the answers obtained from the respondents are relatively similar, (Supplementary Materials Table S1).

### 2.3. Data Collection

The data were collected with a structured questionnaire created in the Survio app. The link to the online survey was shared through social media (Facebook, Instagram and WhatsApp), and by personal contacts of the research group members. The respondents were recruited voluntarily. We also asked the participants to share the study link to increase the number of persons who receive the invitation to the study and thus increase study participants. Before starting the survey, the participants were acquainted with a short description of the research and its purpose. They were also informed of anonymity and confidentiality. The respondents did not provide their names or contact details and were able to complete the survey at any stage of its completion. Responses were saved only after clicking the “send” button after completing the survey. Participants were not rewarded for participating in the study.

### 2.4. Data Analysis

Analyses were conducted in Statistica 12.0 and PQstat. Firstly, the relationships were examined between answers for the period before and during the pandemic with the Wilcoxon test for dependent samples relying on the Z-statistic for *p* < 0.05 and at the α = 0.05 significance level. Based on the resulting data and the analysis of regressive logistic model comparison based on the odds ratio, variables affecting the loss or gain of body mass were determined. Furthermore, the Hosmer–Lemeshow test was conducted to assess the similarity of the observed counts and the predicted probability. Dietary behavior changes and the associated body mass gain included nine independent variables. For that purpose, a logistic regression analysis was conducted. The model quality was not high (R^2^ Pseudo = 0.08, R^2^ Nagelkerke = 0.14, and R^2^ Cox-Snell = 0.11). Simultaneously, the model is statistically significant (*p* < 0.001 of the likelihood-ratio test), and thus the independent variables included in the model are statistically significant. Additionally, a Hosmer–Lemeshow test was also conducted that indicated a lack of significance *p* = 0.162, which is a desirable result as it implies the similarity between the observed counts and the predicated probability.

The Kruskal–Wallis one-way analysis of variance by ranks was then used to verify the hypothesis regarding the insignificance of differences between the medians of the examined variable across several populations (Poland, Austria and UK). Additionally, Dunn’s test was used which accounts for tied ranks and corrects for multiple comparisons. 

## 3. Results

### 3.1. Socio-Demographic Characteristic

The study encompassed a total of 1071 adults from Poland (*n* = 407), Austria (*n* = 353) and the United Kingdom (*n* = 311). The study sample included 604 women and 467 men. Full socio-demographic data are shown in Table 1.

### 3.2. Meal Frequency and Shopping

More respondents reported eating 4–5 meals per day with greater frequency. During the pandemic and the recommended self-isolation, the general shopping frequency decreased, regardless of the place and manner. The respondents shopped less frequently at the supermarkets and local stores, as presented in Table 2 (for detailed statistics see Supplementary Materials Table S2). However, there has been an increased interest in online grocery shopping. 

### 3.3. Dietary Habits

In Table 2, studied dietary habits has been presented. The respondents ate self-prepared meals more frequently. A statistically significant difference in the frequency of ordering readymade meals from restaurants or catering companies has been observed *p* < 0.05. Namely, the frequency of ordering readymade meals from restaurants or catering companies largely increased during the pandemic. However, a partial or total lockdown limited the potential for traditional restaurant attendance, as reflected by the current findings. Additionally, statistically significant difference in the frequency of eating out was observed. During the pandemic, the frequency of eating out dropped significantly regardless of the country.

### 3.4. The Frequency of Consumption of Particular Foods

The resulting data revealed an increased frequency of the daily consumption of basic food products such as dairy, grains, fats and vegetables *p* < 0.001 (see Table 3 and Supplementary Materials Table S3.). In the case of meat and fruit, the result was not statistically significant. Notably, the frequency of eating sweets and snacking increased across the entire analyzed population. A rise in the frequency of purchasing frozen goods and long-term food storage products was also observed. The respondents declared that during the COVID-19 confinement, they have been drinking tea and water more frequently and coffee and juices less frequently. 

### 3.5. Risk of the Body Mass Gain or Loss

**Figure 1:**
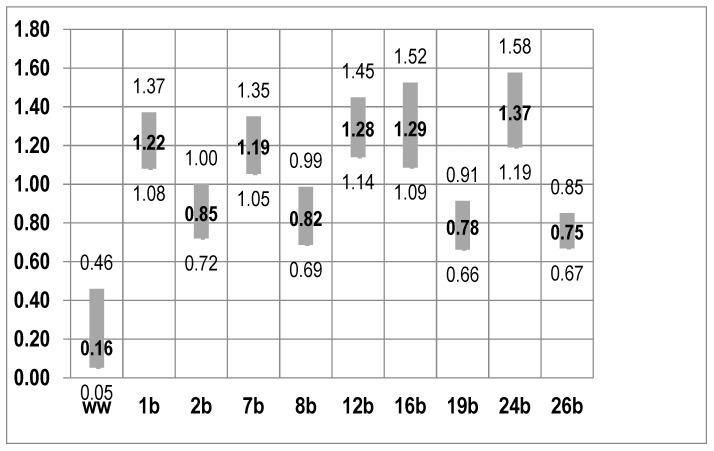
Factors determining weight loss: logistic regression based on the odds ratio +95%Cl; ww—absolute term; 1b—consumption of 4–5 meals a day; 2b—frequency of shopping; 7b—ordering ready-made meals at restaurants; 8b—frequency of eating out; 12b—frequency of eating sweets; 16b—frequency of eating fruits; 19b—frequency of eating fish and seafood; 24b—frequency of drinking alcohol; 26b—frequency of physical activity.

Some dietary habit modifications provoked body mass changes. The current study presents a simulation of factors that could affect the loss or gain of body mass. In the case of the model comparison analysis, the optimal model examining the impact of the COVID-19 pandemic. The odds of gaining body mass during the pandemic depend on the listed variables in the manner determined by the odds ratio (Figure 1).

The consumption of 4–5 meals a day (*p* = 0.001), ordering readymade meals at restaurants (*p* = 0.005), the frequency of eating sweets (*p* = 0.000), fruits (*p* = 0.003) and drinking alcohol (*p* = 0.000) increases the odds of gaining body mass. Meanwhile, a higher frequency of shopping decreases the odds of gaining body mass *p* = 0.049. Furthermore, it was observed that eating out may limit the odds of gaining body mass *p* = 0.034, just like regular physical activity *p* = 0.000.

**Figure 2:**
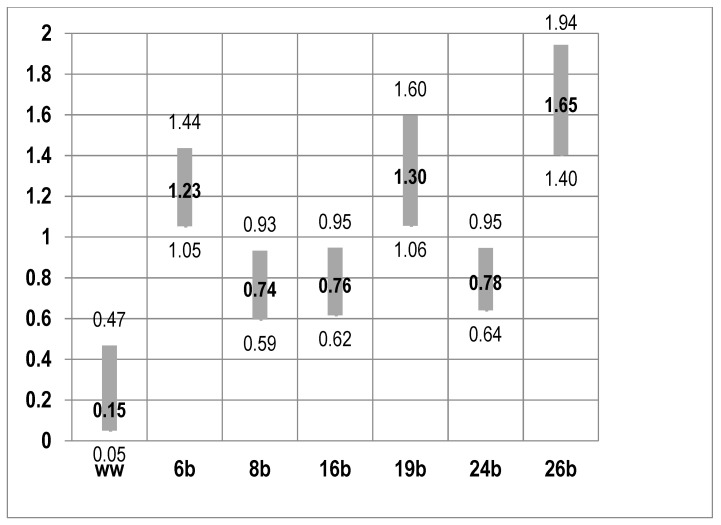
Factors determining weight loss: logistic regression based on the odds ratio +95%Cl; ww—absolute term; 7b—frequency of preparation of home-made meals; 8b—frequency of eating out; 16b—frequency of eating fruits; 19b—frequency of eating fish and seafood; 24b—frequency of drinking alcohol; 26b—frequency of physical activity.

A similar model framework presented the odds of losing body mass during the pandemic (Figure 2). 

The model identifies six independent variables which increase the odds of losing body mass during the pandemic. The factors include the preparation of homemade meals, which increased markedly during the COVID-19 pandemic. The model of homemade meal consumption *p* = 0.009 increases the odds of losing body mass. The consumption of fish and seafood *p* = 0.013 and heightened physical activity *p* < 0.001 have a similar effect. Additionally, increasing the frequency of eating out *p* = 0.01, consuming fruit *p* = 0.013 or alcohol *p* = 0.011 may worsen the odds of losing body mass. 

**Figure 3:**
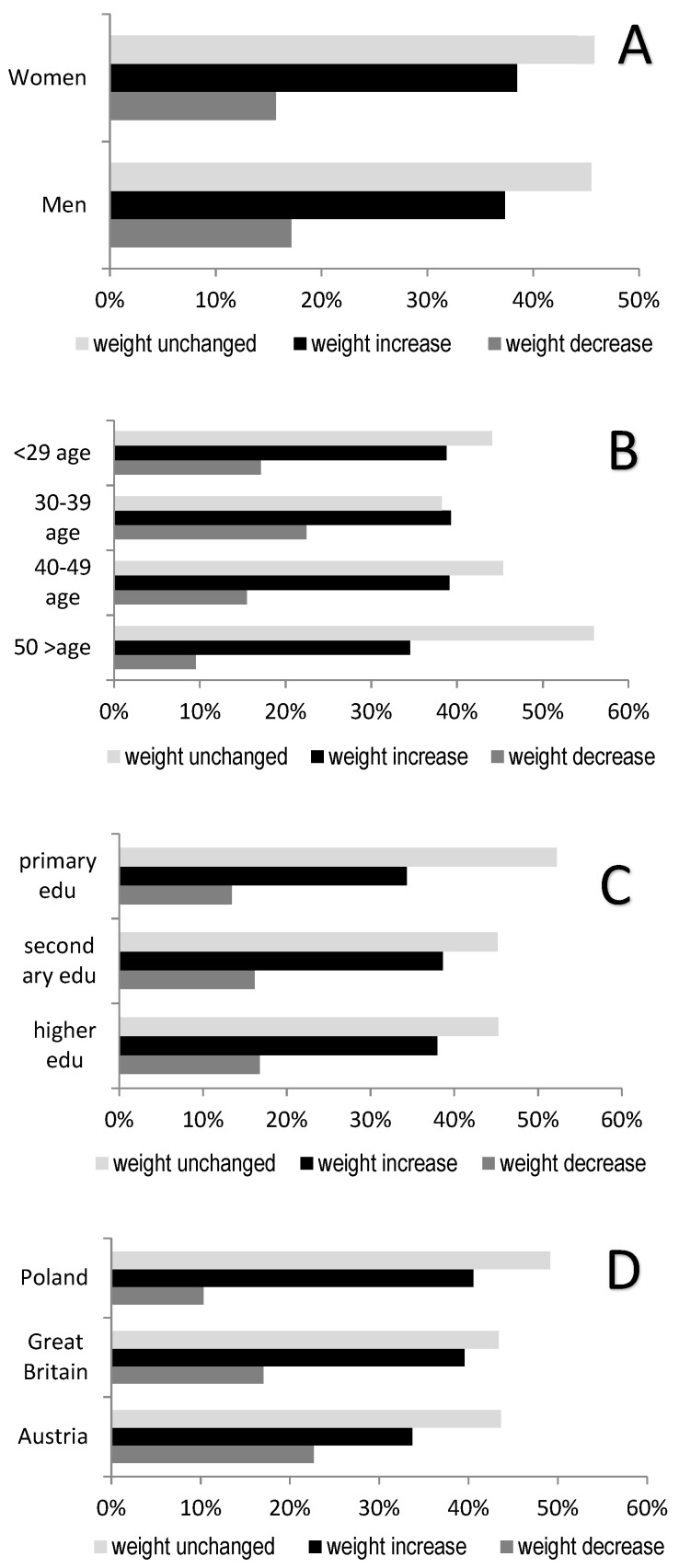
Reported trends in weight change during COVID-19 pandemic. Comparison between groups χ^2^ Pearson’s (**A**) gender, *p* = 0.800; (**B**) age, *p* = 0.000; (**C**) education, *p* = 0.846; (**D**) country, *p* = 0.043; *n* = 1071.

The analysis of body mass changes during the pandemic revealed that the largest number of people had reported no impact of the isolation and the numerous restrictions on body mass change (Figure 3). A comparison of the responses provided by men and women yielded no statistical significance. However, it is worth noting that 38.51% of women and 37.34% of men self-reported increased body mass. Education level also had no impact on the findings concerning body mass change during the pandemic. However, differences were observed related to the age and country of origin of the respondents. The group that responded the least dynamically to the epidemic situation included people over 50 years of age. In this group, 55.95% of the respondents observed no body mass changes. In terms of the country of origin, the largest percentage of people who declared body mass gain came from the United Kingdom and accounted for 39.86% of the total.

### 3.6. Nutritional Behavior of Consumers Depending on the Country of Residence

Statistical analysis showed that the studied eating habits did not differ significantly between Austria and UK. On the other hand, differences were found between Poland and the two countries what can be observed in (Supplementary Table S4).

The first question regarding the daily number of meals before the COVID-19 pandemic revealed no differences between the respondents from the examined countries. 

The observed difference during COVID-19 concerned the more frequent consumption of meals in Poland compared to Austria *p* = 0.03. 10.81% of the Polish respondents declared eating a hot meal several times per day, whereas 78.62% reported once a day. Austrians and the British rarely (1.29%; 3.12%) reported eating a hot meal several times a day, but at least one hot meal is consumed by a majority (64.02%; 65.60%). A post hoc Dunn’s analysis with Bonferroni adjustment indicates that significant differences between Poland and the United Kingdom, as well as Poland and Austria. Dietary behaviors presented by Polish respondents indicate that as many as 40.30% prepared homemade meals before the pandemic every day, while in Austria and the United Kingdom, that percentage was at the level of 16.99% and 20.90%, respectively. During the COVID-19 pandemic, the situation slightly changed, as the percentage of people preparing homemade meals every day increased to 42.21% for Austria and 45.02% for the UK, compared to 75.68% for Poland (*p* < 0.005).

Before the lockdown, most people self-reported going shopping several times a week (Poland 61.67%; Austria 55.52%; UK 54.98%, Poland vs. Austria and UK *p* = 0.000). During the pandemic people self-reported going shopping several times a week (Poland 46.44%; Austria 40.23%; UK 43.73%) or several times a month (Poland 41.28%; Austria 34.84%; UK 33.12%; Poland vs. Austria and UK *p* = 0.000).

It has been found that shopping at the supermarket before and during pandemic has been more popular in Poland than in the other countries, because as many as 41.03% of the respondents self-reported shopping at discount stores several times a week. Contrastingly, the residents of Austria and the UK shop at large-area stores more rarely (25.21% and 20.90%, respectively; Poland vs. Austria and UK *p* = 0.000). Before COVID-19, a very popular practice was to shop at local suppliers and food markets, especially among the residents of Austria 40.51% and the UK 46.30%, who reported shopping in this manner several times a week. Since Poles use the local markets less frequently *p* = 0.000.

Before the pandemic, 73% of Austrians, 80% of British and 74% of Poles declared that they have never purchased groceries online or have done so sporadically. A statistical analysis of variance revealed no statistical difference in the frequency of online shopping before the pandemic between countries *p* = 0.08. During the pandemic online shopping is used by far more people than before. 42.49% of the respondents from Austria, 38.91% from the UK and 41.52% from Poland shop online at least several times a month.

Many consumers reported eating readymade meals ordered from restaurants with home delivery. Before the pandemic, the difference in answers was statistically significant (Poland vs. UK and Austria; *p* < 0.000). Eating readymade meals several times a week was reported by 21.25% residents of Austria and 19.94% of the United Kingdom, against only 6.14% of respondents from Poland. Meanwhile, 30.47% of the Polish respondents never ordered home readymade meals, whereas in Austria that percentage accounts for 19.55% of the study sample and in the UK for 16.08% (Poland vs. UK and Austria; *p* < 0.000).

Highly dynamic changes were observed in the frequency of eating out. Residents of Austria and the UK often ate out before the COVID-19 pandemic. The largest number of answers indicated eating out several times a week; 35.69% in Austria and 30.55% in the UK. Meanwhile, only 8.60% of the Polish respondents declared eating out several times a week.

The responses inform that during the pandemic, the Poles used restaurants most frequently (20.15% reported several times a month), compared with 14.16% in Austria and 11.58% in the UK. Additionally, 56.66% of the residents of Austria and 59.49% of the UK have stopped eating out entirely during COVID-19, against 43.24% in Poland (*p* < 0.05).

### 3.7. Frequency of Consuming Selected Food Product Groups in Studied Countries

#### 3.7.1. Frozen Goods

Before the lockdown, there were no statistically significant differences in the frequency of consuming frozen goods between Austria, UK or Poland (see Table S5). The largest number of answers indicated that the respondents bought these products several times a month (Poland 39.80%; Austria 35.13%; the UK 46.62%) or less frequently (Poland 38.33%; Austria 39.38%; the UK 31.51%). The Sars-Cov-2 pandemic caused people to shop less frequently and store frozen goods or long-term storage products. Frozen goods have been purchased more eagerly than before the pandemic *p* = 0.03. Significant differences concern Poland and the United Kingdom, as well as Poland and Austria, which resembles data for preserves. The increase in preserves consumption during pandemic has been observed in all of the studied countries. There has been a significant increase in UK in relation to Austria *p* = 0.019 and Poland to UK *p* = 0.004.

#### 3.7.2. Sweets and Snacks

Before the pandemic, the consumption of sweets was high. In Poland, as many as 36.86% of the respondents declared eating sweets several times a week. Slightly lower levels of consumption were declared by respondents from Austria 27.48% and the UK 21.22%. Furthermore, there was a group which consumed sweets before the pandemic several times a day: Poland 3.93%, Austria 5.67% and the UK 3.59%. Residents of the United Kingdom reported eating sweets much less frequently than respondents from Poland *p* = 0.000. In the face of the pandemic, the consumption of sweets increased in all countries. However, there were no statistical differences between the three examined samples. The largest number of respondents reported eating sweets several times a week also during the pandemic.

#### 3.7.3. Eggs and Dairy Products

The pandemic brought about an increase in the consumption of eggs and dairy. Before the lockdown, the respondents reported eating dairy and eggs several times a week: Austria 47.88%, the UK 53.38% and Poland 42.51%. The obtained results indicated no relationship between these countries in the frequency of dairy consumption. Meanwhile, during the pandemic, the frequency of dairy consumption in Poland remained unchanged, whereas the consumption of eggs and dairy in Austria and the UK increased (Poland vs. UK; *p* = 0.000). 

#### 3.7.4. Grains

The questionnaire results indicated an increase in grain consumption during the pandemic. The answers for the period before the pandemic were statistically significant, with the differences concerning answers provided by the residents from Poland and the two other countries, Austria and the UK. During the pandemic, grain consumption increased, and the differences between the countries were the same as in the period preceding the pandemic. 

#### 3.7.5. Oils

Even though (Table S3) indicates an increase in fat and edible oil consumption, no differences were revealed for the period either before or during the pandemic. 

#### 3.7.6. Fruits and Vegetables

In terms of fruit consumption, no statistically significant differences were revealed between the two examined periods (see Table S2), but the differences between the countries were statistically significant. Regardless of their place of residence, most respondents declared eating fruit several times a week before COVID-19. A similar situation occurred during the lockdown (Austria 53.82% versus 52.69%; the UK 59.16% versus 59.81%; Poland 37.84% versus 52.69%). Statistical data analysis regarding the frequency of fruit consumption revealed that the differences concerned Poland and Austria, as well as Poland and the UK. Austrian and British respondents consume fruit more frequently than Poles, both before the pandemic and during the COVID-19 isolation. 

Moreover, statistical analysis indicated significant differences *p* = 0.000 in vegetable consumption both before and during the pandemic. The post hoc Dunn’s analysis indicated that the differences concerned Poles and Austrians, as well as Poles and the British. Both before and during the pandemic, consuming vegetables several times a week was reported by respondents from Austria: 39.60% versus 50.99%, the UK: 28.94% versus 48.55% and Poland: 25.31% versus 27.52%. Poles consumed fruit much less frequently both before and during the isolation.

#### 3.7.7. Meat

The largest number of people declared that Poland eats meat several times a week 52.58%; Austria 49.01% and the UK 49.84%. The sample included people who refrained from eating meat at all: Poland 5.9%; Austria 6.52%; the UK 2.89%. The statistic shows an increase in the consumption of meat and meat products in all the examined populations, but also an increased percentage of people refraining from eating meat entirely: Poland 7.13%; Austria 7.37% and the UK 3.27%.

Results indicate a significant difference (*p* = 0.000) between the compared countries in the frequency of meat consumption before and during the pandemic and also between Poland vs. Austria and UK (*p* = 0.000). 

#### 3.7.8. Fish and Seafood

The largest percentage of people consuming fish and seafood before the pandemic reported eating these goods several times a month, with the Poles consuming them most rarely. Statistical analysis indicated differences in the answers between Poland and Austria (*p* = 0.000), as well as Austria and the United Kingdom (*p* = 0.01). Similar differences between the countries were not demonstrated during the pandemic. The pandemic period effectively limited the consumption of fish and seafood in all groups of respondents.

#### 3.7.9. Beverages and Alcohol

Before the pandemic, coffee was consumed several times a day by 43.24% of Poles, 23.23% of Austrians and 12.86% of British, and at least once a day by 33.66% of Poles, 39.38% of Austrians and 41.48% of British. During the pandemic, the general frequency of coffee consumption fell in all three groups, but some preferences regarding coffee consumption remained unchanged. Consequently, there has been still an observable statistically significant difference in the frequency of coffee consumption depending on the place of residence. 

Before the pandemic, 35.37% of the respondents from the UK, 32.19% from Poland and 26.91% from Austria drank tea several times a day. Frequency trends remained unchanged, because the differences between Poland and the UK, as well as the UK and Austria, were observed for the periods both before and during the pandemic. The frequency of drinking tea by the British was statistically higher.

In terms of water consumption, no statistically significant differences were observed between the countries in any time period. Meanwhile, research shows that the consumption of juices dropped. A statistical analysis revealed that the differences in the consumption of juices and sweets drinks before the pandemic concerned mainly Poland and the other two countries, the UK and Austria. Meanwhile, during the pandemic, the differences concerned only the residents of Poland and the UK (*p* = 0.006) 

Before the pandemic, an average of 45% of the respondents declared consuming alcohol several times a month. Approximately 14% consumed alcohol more frequently, i.e., several times a week. However, the pandemic caused 22% of the respondents to declare including alcohol in their diet several times a week. No statistically significant differences were observed between the countries either for the pandemic period or for the preceding timeframe

### 3.8. Physical Activity

Physical activity together with well-balanced diet is a component of a rational lifestyle, which is why the reduction in various forms of activity during COVID-19 presents a cause for concern. Statistical analysis indicated a reduced daily frequency of physical activity during the pandemic (*p* = 0.000). More respondents reported doing sports less frequently (Table 4).

Before the pandemic, the largest group of respondents declared doing sports several times a week or several times a month. However, the time of the pandemic and the restrictions reduced the frequency of practicing any kind of physical activity. 

Meanwhile, during the lockdown, a significant difference in the frequency of physical activity between the compared countries was observed. The analysis indicates that significant differences also concern Poland and the United Kingdom (*p* = 0.028).

## 4. Discussion

The perceived diet-related behavioral changes induced by the social isolation of COVID-19 were examined on a large sample of adults in Poland, Austria and the United Kingdom. From the epidemiological standpoint, it is important to determine how healthy habits are affected by the time of the restrictions and define the consequences of such changes for human health. 

### 4.1. Dietary Habits

During the pandemic, daily consumption of 4–5 meals was declared by 12.18%, 13.18% and 0.74% more respondents from Austria, the United Kingdom and Poland, respectively. It may be related to the fact that many respondents followed at least partially a remote working model, which facilitated the preparation and consumption of a larger number of meals. The European Commission estimated that nearly 40% of active workers switched to remote work only because of the pandemic. Furthermore, the tendency to work remotely is expected to intensify, which additionally exposes the importance of analyzing changes in dietary habits [17,18]. 

Furthermore, consumers were observed to reduce the frequency of grocery shopping. The largest drop occurred in Poland, where the number of people reporting shopping at the supermarket at least once a week decreased by as many as 17.94%. For Austria and the United Kingdom, that difference was 3.40% and 4.50%, respectively. Such indications may be the product of the fear of large concentrations of people, associated with larger stores [19]. The pandemic increased the importance of online shopping, although the interest in that form of making groceries varied across the examined countries. During the pandemic, the percentage of Polish respondents shopping online at least once a week rose by only 2.46%. Austrians and the British participating in the study reported shopping online, respectively, 14.16% and 11.58% more frequently than before the pandemic. These numbers are far lower than the findings obtained in a study of Qatari consumers where 35.35% reported shopping online for more groceries and at a greater frequency [20]. On the other hand, in Tunisia, only 2.1% of the consumers used the opportunity to shop on the Internet. The interest in online shopping varies across the globe and results from a plethora of factors, such as the availability of fresh goods, restrictions affecting the opening hours of certain stores or stalls or their potential freezing.

Social distancing was also manifested in the drastically reduced frequency of eating out. This change resulted from the fear of potentially contracting the virus, but also the closure of many foodservice establishments, which either stopped operating entirely or offered only takeaway and home delivery [21]. The greatest differences in eating out at least once a week were observed in Austria and the United Kingdom, which recorded drops of 46.74% and 40.84%, respectively. In Poland, that value decreased by 9.58%, but one should consider that Polish respondents reported eating out before the pandemic over 30% less than Austrians or the British

The number of people ordering in from food service establishments may differ significantly depending on the cultural background, level of income and, paradoxically, the fear of the virus. Still, such concerns are not unfounded; it was demonstrated that more than 60% of infections at a public hospital in Hanoi were related to the deliveries of readymade meals [22]. A study on Spanish adults revealed that as many as 45.7% of the respondents declared more frequent meal preparation on their own. Interestingly, upon the analysis of other dietary habit changes, the examined group exhibited more favorable food behaviors [3]. A greater number of homemade meals may bring immense health benefits since it allows one to fully control the ingredients of each meal. [23]. Furthermore, the preparation of homemade meals has more advantages than the potential benefits related to physical health. Mills et al. (2020) indicated that cooking at home was related to social and emotional benefits. It is a highly desirable phenomenon, particularly in the pandemic era, when many people are under severe stress and experience a lack of control over the surrounding reality [24,25].

### 4.2. Food Products and Beverages Consumption

During the pandemic, consumers in the examined countries started to stockpile long-term storage products.

Similarly, Italian consumers were observed to increase their consumption of preserves, frozen goods and products such as noodles, flour, eggs and pasteurized milk [26]. In Tunisia, 26.3% of study participants confirmed that they were more eager to choose food products with a longer use-by date [19]. In Qatar, the consumption of frozen goods and canned goods increased by more than 20% [20]. The tendency to stock up on food was also observed in China. However, Chinese consumers mostly augmented their reserves of fresh foods from 3.37 to 7.37 days and were inclined to pay as much as 60.47% more for these products [27,28].

Negative changes observed in the wake of the COVID-19 pandemic-related restrictions include primarily reduced sales of fresh goods. Decreased consumption of fish and seafood was observed by many authors [4,29,30,31], along with decreased consumption of fruit and vegetables by others [3,32,33]. The latter researchers asserted that such a major change (fruit had a drop by 24.9%; vegetables had a drop by 19.5%) may be caused by the physical absence of many people at work or the reduced availability of these products [3].

In our study, significant changes were observed for sweets (a rise), fats (a rise), vegetables (a rise) and fish and seafood (a drop). Regarding fruit and meat, no significant changes were observed. Scarmozzino and Visioli (2020) demonstrated greater consumption of fresh fruit and vegetables in the case of 21.2% of the respondents. Simultaneously, 8.7% of people reduced their consumption of fruit and vegetables, ascribing that change to the difficulties in finding open grocery stores. Meanwhile, Laguna et al. (2020) presented a broader range of products, whose consumption in Spain changed for the better during the pandemic. The respondents increased their frequency of purchasing eggs, milk, fresh vegetables and fruit (particularly citrus fruit), legumes, oil and frozen vegetables, and reduced the frequency of purchasing readymade meals, desserts, breadstuff, snacks, chocolate and cold cuts [30,34]. Data obtained by Di Renzo et al. (2020) and Bracale and Vaccaro (2020) indicate increased purchases of ingredients necessary for the preparation of homemade baked goods (flour, yeast and cake ingredients) and, by the same token, increased consumption of meals from home recipes, which was also corroborated by the authors’ own research [4,32].

In Poland, Górnicka et al. (2020) observed a rise in the consumption of vegetables (18.8%), fruit (15.2%), full-grain products (16.3%), low-fat meat, eggs (15.7%) and milk and milk products (20.8%) [5]. Our findings are in concordance with these studies, we also noticed the consumption of vegetables, dairy and eggs and grain products increased.

Positive changes in dietary habits were also demonstrated outside Europe. Hassen et al. (2020) observed that Qatari residents increased the consumption of fruit and vegetables by as much as 32.4%, healthy foods by 32.3% and healthy snacks by 20.9%. In the case of sweets, cakes, cookies and confectionery products, 28.7% of the respondents declared reduced consumption, whereas 24.6% reported increased consumption [20]. In light of these observations, it is interesting to consider the findings of Husain et al. (2020). Research conducted in Kuwait indicated that before the pandemic, 49% of the respondents consumed fast foods more frequently (1–2 times a week) and, during the pandemic, 82% refrained from eating such foods entirely [29]. Similarly, in Brazil, Steele et al. (2020) indicated a statistically significant, though small, increase in the frequency of consuming vegetables, fruit, beans and other legumes. In the entire cohort of participants, the indicators of unhealthy food habits have shown virtually no change in the face of the new situation [35]. Chang et al. (2020) demonstrated that in the case of the analyzed sample of respondents from China, the largest sales growth was recorded for grains (13.1%), followed by fruit and vegetables (9.6%). Meanwhile, no statistically significant changes were observed for other food product [36]. 

The current research revealed a significant increase in the consumption of snacks and sweets. The results of other authors vary and remain inconclusive. Some authors also report (as in the current study) an increased consumption of sweets [4,33,34], while others noted a drop in the consumption of mainly chocolate, desserts and salty snacks [3,32].

The results of our study revealed that the general frequency of consuming coffee and juices dropped in all of the three analyzed countries. In turn, the consumption of tea increased, even though the trends regarding the frequency of tea consumption remained unchanged. The type of liquids consumed during the day is a highly individual matter determined by a range of cultural, social and economic factors and the pandemic situation had a varied effect on the dietary behaviors related to daily drink consumption. 

Importantly, the frequency of alcohol consumption increased in all of the examined countries. During the pandemic, alcohol consumption at least once a week rose by 11.05%, 12.22% and 6.88% for Austria, the United Kingdom and Poland, respectively. Other authors also noted this disquieting change. In Poland, Górnicka et al. (2020) observed increased alcohol consumption in 18.1% of the respondents and Sidor et al. (2020) in 14.6% [5,37]. In turn, Giacalone (2020) reported that the consumption of alcoholic drinks in Denmark rose by as much as 30.3% [33]; an increased consumption of high alcohol content drinks was also reported in Lithuania [38]. This phenomenon may be the effect of the poor psychological condition of people forced to self-isolate. Isolation may have induced a feeling of helplessness and a lack of control over their situation, which amplifies stress—one of the factors inclining some consumers to drink alcohol [25]. It may be due to the synergistic effects of unemployment, stress from childcare or additional caregiving responsibilities. Zhang et al. (2020) demonstrated that, apart from the consumption of vitamin C, probiotics and other health products, the use of alcohol is one of the reported mechanisms of coping with the fear of the pandemic [31].

On the other hand, some reports point to a major drop in the frequency of alcohol consumption. Such a relationship was observed by Laguna (2020) and Rodriguez-Perez (2020) for the Spanish population, and Di Renzo (2020), as well as Scarmozzino and Visoli (2020), for the Italian population [3,4,30,34]. In both countries, daily consumption of low-alcohol drinks was higher than in the other European countries due to their traditional consumption during meals and frequent meetings with family and friends. Therefore, major drops observed in these countries reflect the limitation of social contacts, which were very often associated with alcohol use. 

### 4.3. Body Mass Changes

Many authors have attempted to reveal a relationship between food habits before the pandemic and the changes that transpired during it. It turns out that people exhibiting fewer healthy food habits responded negatively to the restrictions related to the pandemic (body mass gain, reduced physical activity, etc.), whereas people with more favorable dietary patterns either did not change their habits during the pandemic or took even better care of this aspect of their lives [3,33,37].

The impact of the COVID-19 crisis on weight-related behaviors, including healthy dietary habits and physical activity, is unclear but may be significant [39]. Small body mass changes over relatively short periods may become permanent and, with time, lead to a significant body mass gain. Considering that the current situation may continue for at least several months, this prolonged home confinement may exacerbate the problem of obesity in adults, significantly contributing to the yearly body mass gain [40]. The current study revealed that the largest group of people reported an increase in body mass during pandemic (Figure 3). Gender had no impact on the differences in body mass changes of the respondents. Nearly 40% of men and women declared a body mass gain. A similar relationship among women was reported by Drywień et al., 2020, in which body mass gain was reported by 38% of the examined women and 44% of the obese women before the pandemic [41]. Furthermore, as many as 74% of underweight women lost weight during the pandemic. The findings may suggest that many unsuitable food behaviors may escalate during the pandemic. These findings are consistent with those obtained by Fernandez-Rio at al., who aimed to assess body mass changes of people in Spain during home confinement, and according to the results, most respondents declared no change (0 kg). Men and people with obesity exhibited greater body mass volatility, whereas the elderly showed greater stability [42]. Most of all, the problem of body mass gain concerned people under 50 years of age. Dietary habits of people under 50 may be less ingrained than those of the older ages, which makes them most susceptible to change during the pandemic. Additionally, it should be noted that part of the consumers included in the 50+ age category included people who were no longer professionally active. Thus, the pandemic did not affect their daily life as intensely as in the case of people who had to change their working model or their entire work organization (new security procedures, etc.). In Lithuania, it was observed that approximately one-third (31.5%) of the respondents gained weight and that this tendency was more common in people who were already overweight. An analysis of the multi-factor logistic regression demonstrated that greater chances of body mass gain were related to women of older ages [38], which was not conclusively confirmed by the current findings. Interesting results were obtained by authors conducting research in Hubei Province, China. It was determined that people of normal body mass, who normally experience no problems with excess weight or obesity, exhibited weaker awareness of body mass gain than persons with BMI ≥ 24. In semi-lockdown conditions, they had a tendency to gain body mass [43].

### 4.4. Physical Activity

One of the unsettling elements observed during the pandemic, other than the food-related aspects, is a change in physical activity (PA). Our research shows that long-term isolation led to reduced PA and an intensification of the sedentary behaviors. There is a great deal of evidence justifying the adoption of PA promotion as a global priority in the area of public health during the COVID-19 pandemic [44]. Unfortunately, the pandemic limited the number of places where people could practice physical activity, since many countries prevented or limited access to common places of recreation, both indoors and outdoors. This situation found a reflection in the current research, which indicated a general reduction of physical activity during the time of COVID-19 restrictions in all the examined countries. The current findings complement similar studies on the role of physical activity in the United Kingdom [45,46], Austria [47] and Poland [38,48]. A drop in physical activity during lockdown is corroborated by the findings of other authors who analyzed the levels of PA in other countries [49,50]. According to Ong [51], physical activity dropped by an average of 42% and the least active group accounting for ~51% of the sample included younger and mainly single people. This group, which typically shows lesser activity, exhibited the largest drop of PA during the lockdown. These findings are corroborated by the research [52], conducted on the Spanish population of adults. The study demonstrated that youth, students and highly active men especially reduced their daily reported PA during the COVID-19 lockdown. Caputo et al. [53] wrote an extensive review paper on the relationship between physical activity and the COVID-19 lockdown. Most evidence points to a drop in PA due to social distancing measures, even though in some studies from Italy [4] and Spain [54], the participants generally reported an increase of activity during the lockdown. Good physical condition may also help reduce the mental health pressures related to the outbreak of COVID-19, which is an additional argument for the promotion of physical activity and sport at all ages during the isolation caused by the coronavirus.

## 5. Conclusions

The dietary habits in the studied countries have changed as a result of the epidemic situation. They contribute to the aggravation of the problem of excess body weight and its health consequences. It is extremely important to take action in the area of maintaining a proper diet and physical activity early.

Therefore, it is important to define the most urgent scientific questions that may be translated into policy and practices which positively impact population health in the context of a prolonged pandemic and upon its end. It is a matter of the utmost importance to abandon the negative habits acquired during a lockdown after the pandemic situation has been resolved. However, it is equally important to entrench the positive food-related behaviors developed during self-isolation, particularly those related to meal frequency, quality and the manner of preparation.

## Figures and Tables

**Table 1:** Socio-demographic characteristics of 1071 adults who filled out the questionnaire.

Variables	Sample *n*/%	Variables	Samples *n*/%
Gender		Education	
Women	604/56.40	Primary	77/7.19
Men	467/43.60	Secondary	442/41.24
Other	0/0.00	Degree	552/51.57
Age		Country	
>18	264/24.65	Poland	407/38.00
30–39	285/26.61	Austria	353/32.96
40–49	269/25.11	United Kingdom	311/29.03
>50	253/23.62		

**Table 2:** The frequency and place of the shopping of selected products before and during lockdown.

Question	Total *n* = 1071 (100%)		Poland *n* = 407 (100%)		Austria *n* = 353 (100%)		United Kingdom *n* = 311 (100%)	
	Before	During	Before	During	Before	During	Before	During
Frequency of consumption of 4–5 meals a day/every day *n*/%	311 (29.04)	398 (37.16)	144 (35.38)	147 (36.12)	103 (29.18)	146 (41.36)	64 (20.58)	105 (33.75)
Eating at least one warm meal/at least once a day *n*/%	809 (75.54)	927 (86.56)	364 (89.43)	371 (91.16)	237 (67.14)	299 (84.71)	208 (66.88)	257 (82.63)
Preparing homemade meals/at least once a day *n*/%	378 (35.29)	579 (55.74)	253 (62.16)	308 (75.68)	60 (16.99)	149 (42.21)	65 (20.90)	140 (45.02)
Shopping frequency/at least once a week *n*/%	123 (11.49)	87 (8.13)	64 (15.72)	28 (6.88)	30 (8.5)	34 (9.63)	29 (9.32)	25 (8.04)
Shopping in the supermarket/at least once a week *n*/%	342 (31.93)	243 (22.68)	179 (43.98)	106 (26.05)	96 (27.16)	84 (23.80)	67 (21.54)	53 (17.04)
Shopping in local marketplace/at least once a week *n*/%	499 (46.59)	413 (38.57)	168 (41.29)	149 (36.61)	168 (47.59)	146 (41.36)	163 (52.69)	118 (37.95)
Shopping online/at least once a week *n*/%	30 (2.79)	126 (11.77)	13 (3.20)	23 (5.66)	8 (2.27)	58 (16.43)	9 (2.89)	45 (14.47)
Ordering readymade meals at restaurants/at least once a week *n*/%	191 (17.84)	264 (24.66)	34 (8.35)	43 (10.56)	83 (23.51)	103 (29.18)	74 (23.80)	118 (37.94)
Eat out/at least once a week *n*/%	361 (33.70)	30 (2.80)	47 (11.55)	8 (1.97)	175 (49.57)	10 (2.83)	139 (44.70)	12 (3.85)

*n*—number of participants, all data not normally distributed were tested with the chi-square test. Significant *p*-values (<0.05).

**Table 3:** The frequency of the consumption of selected products before and during lockdown.

Products	Total *n* = 1071 (100%)		Poland *n* = 407 (100%)		Austria *n* = 353 (100%)		United Kingdom *n* = 311 (100%)	
	Before	During	Before	During	Before	During	Before	During
Frozen food/ at least once a week *n*/%	86 (8.03)	128 (11.95)	17 (4.18)	25 (6.14)	40 (11.33)	45 (12.74)	29 (9.32)	58 (18.65)
Canned food/ at least once a week *n*/%	70 (6.54)	108 (10.08)	11 (2.70)	22 (5.41)	27 (7.65)	34 (9.63)	32 (10.29)	52 (16.72)
Sweets and snacks/ at least once a day *n*/%	172 (16.06)	232 * (21.67)	68 (16.71)	89 (21.66)	63 (17.85)	86 (24.36)	41 (13.19)	57 (18.43)
Diary and eggs/ at least once a day *n*/%	430 (40.15)	516 (48.18)	164 (40.29)	165 (40.54)	142 (40.23)	176 (49.86)	124 (39.87)	175 (56.27)
Cereal products/ at least once a day *n*/%	764 (71.33)	804 (75.07)	242 (59.46)	252 (61.92)	282 (79.89)	296 (83.86)	240 (77.17)	296 (82.31)
Fats and oil/ at least once a day *n*/%	563 (52.57)	595 (55.55)	240 (58.97)	246 (60.45)	160 (45.32)	180 (50.99)	163 (52.41)	169 (54.34)
Fruits/ at least once a day *n*/%	373 (34.83)	397 (37.07)	196 (48.16)	208 (51.11)	110 (31.16)	115 (32.58)	67 (21.54)	74 (23.79)
Vegetables and nuts/ at least once a day *n*/%	709 (66.20)	777 (72.55)	238 (58.48)	251 (61.67)	251 (71.10)	279 (79.04)	220 (70.74)	247 (79.42)
Meat/ at least once a day *n*/%	340 (31.74)	358 (33.43)	131 (32.21)	126 (30.95)	109 (30.88)	114 (32.29)	100 (32.15)	118 (36.95)
Fish and seafood/ at least once a week *n*/%	235 (21.94)	225 (21.01)	70 (17.20)	67 (16.46)	113 (32.01)	98 (27.76)	52 (16.72)	60 (19.29)
Coffee/ at least once a day *n*/%	703 (65.64)	685 (63.96)	313 (76.90)	310 (76.17)	221 (62.61)	220 (62.32)	169 (54.34)	155 (49.84)
Tea/ at least once a day *n*/%	626 (58.45)	686 (44.73)	235 (57.74)	248 (60.93)	191 (54.11)	206 (58.36)	200 (64.31)	232 (74.59)
Water/ at least once a day *n*/%	933 * (87.12)	949 * (88.61)	356 (87.47)	361 (88.70)	312 (88.39)	318 (90.08)	265 (85.21)	270 (86.82)
Juice and sweets drinks/ at least once a day *n*/%	181 (16.90)	174 * (16.25)	60 (14.74)	64 (15.73)	67 (15.77)	55 (13.91)	54 (17.36)	55 (17.69)
Alcohol/ at least once a week *n*/%	160 (14.94)	265 (24.75)	66 (16.22)	94 (23.09)	42 (11.89)	81 (22.95)	52 (16.72)	90 (28.94)

*n*—number of participants, all data not normally distributed were tested with the chi-square test. Significant *p*-values (<0.05), no significant differences are marked * *p* > 0.05.

**Table 4:** Frequency of physical activity.

	Few Times per Day	One a Day	Few Times per Week	Few Times per Month	Once or Less per Month	Never or Almost Never
Before pandemic						
Austria	1.98%	6.52%	37.39%	30.31%	15.30%	8.50%
United Kingdom	1.29%	2.57%	40.84%	22.83%	20.90%	11.58%
Poland	3.19%	13.76%	33.42%	26.54%	13.76%	9.34%
During pandemic						
Austria	1.70%	10.48%	30.31%	24.93%	20.40%	12.18%
United Kingdom	0.97%	5.47%	29.26%	24.76%	25.08%	14.47%
Poland	1.97%	13.02%	28.99%	22.60%	19.66%	13.76%

Austria *n* = 353, UK *n* = 311, Poland *n* = 407, *p* < 0.05; before pandemic chi-square test = 0.002; during pandemic chi-square test =0.11.

## Supplementary Materials

The following are available online at https://www.mdpi.com/article/10.3390/nu13051690/s1, Table S1. Alpha Cronbach test; Table S2. Comparison of adults’ dietary habits before and during COVID-19 pandemic *n* = 1071; Table S3. The frequency of consumption of particular foods before and during COVID-19 for analyzed population *n* = 1071; Table S4. Nutritional behavior and shopping habits of consumers depending on the country of residence; Table S5. Nutritional behavior of consumers depending on the country of residence. Questionnaire S1. Comparison of dietetary behaviours before and during the COVID19 pandemic.

## References

[1] Chowell G., Mizumoto K. (2020). The COVID-19 pandemic in the USA: What might we expect?. Lancet.

[2] Martinez-Alvarez M., Jarde A., Usuf E., Brotherton H., Bittaye M., Samateh A.L., Antonio M., Vives-Tomas J., D’Alessandro U., Roca A. (2020). COVID-19 pandemic in west Africa. Lancet Glob. Health.

[3] Rodríguez-Pérez C., Molina-Montes E., Verardo V., Artacho R., García-Villanova B., Guerra-Hernández E.J., Ruíz-López M.D. (2020). Changes in Dietary Behaviours during the COVID-19 Outbreak Confinement in the Spanish COVIDiet Study. Nutrients.

[4] Di Renzo L., Gualtieri P., Pivari F., Soldati L., Attinà A., Cinelli G., Leggeri C., Caparello G., Barrea L., Scerbo F. (2020). Eating habits and lifestyle changes during COVID-19 lockdown: An Italian survey. J. Transl. Med..

[5] Górnicka M., Drywień M., Zielinska M., Hamułka J. (2020). Dietary and Lifestyle Changes During COVID-19 and the Subsequent Lockdowns among Polish Adults: A Cross-Sectional Online Survey PLifeCOVID-19 Study. Nutrients.

[6] Di Santo S.G., Franchini F., Filiputti B., Martone A., Sannino S. (2020). The Effects of COVID-19 and Quarantine Measures on the Lifestyles and Mental Health of People Over 60 at Increased Risk of Dementia. Front. Psychiatry.

[7] Kishimoto M., Ishikawa T., Odawara M. (2021). Behavioral changes in patients with diabetes during the COVID-19 pandemic. Diabetol. Int..

[8] Brown S., Opitz M.-C., Peebles A.I., Sharpe H., Duffy F., Newman E. (2021). A qualitative exploration of the impact of COVID-19 on individuals with eating disorders in the UK. Appetite.

[9] Snuggs S., McGregor S. (2021). Food & meal decision making in lockdown: How and who has Covid-19 affected?. Food Qual. Prefer..

[10] Marty L., de Lauzon-Guillain B., Labesse M., Nicklaus S. (2021). Food choice motives and the nutritional quality of diet during the COVID-19 lockdown in France. Appetite.

[11] Siłownie, kluby fitness i obiekty wspinaczkowe - Ministerstwo Rozwoju, Pracy i Technologii - Portal Gov.pl. https://www.gov.pl/web/rozwoj-praca-technologia/silownie-i-kluby-fitness.

[12] Austrian Government Latest Information on the Coronavirus Situation in Austria. https://www.austria.info/en/service-and-facts/coronavirus-information.

[13] Local COVID-19 alert level update: 15 October 2020 - GOV.UK. https://www.gov.uk/government/news/local-covid-19-alert-level-update-15-october-2020.

[14] Kwestionariusz FFQ. http://www.uwm.edu.pl/edu/lidiawadolowska/html/ffq6.html.

[15] Bersenkowitsch I., Kogler B., Tritscher A., Visontai S., Putz P. (2019). The Vienna Food Record User-centered Development of A Pro-spective Food Record for Application in Austrian Adults. Ernaehrungs Umschau Int..

[16] https://www.epic-norfolk.org.uk/about-epic-norfolk/nutritional-methods/ffq/.

[17] European Commission Telework in the EU before and after the COVID-19: Where we were, where we head to. https://ec.europa.eu/jrc/sites/jrcsh/files/jrc120945_policy_brief_-_covid_and_telework_final.pdf.

[18] International Labour Organization An Employers’ Guide on Working from Home in Response to the Outbreak of COVID-19. https://www.ilo.org/actemp/publications/WCMS_745024/lang--en/index.htm.

[19] Jribi S., Ben Ismail H., Doggui D., Debbabi H. (2020). COVID-19 virus outbreak lockdown: What impacts on household food wastage?. Environ. Dev. Sustain..

[20] Ben-Hassen T., El-Bilali H., Allahyari M.S. (2020). Impact of COVID-19 on Food Behavior and Consumption in Qatar. Sustainability.

[21] Cranfield J.A.L. (2020). Framing consumer food demand responses in a viral pandemic. Can. J. Agric. Econ. Can. D’Agroeconomie.

[22] Nguyen T.H.D., Vu D.C. (2020). Food Delivery Service During Social Distancing: Proactively Preventing or Potentially Spreading Coronavirus Disease–2019?. Disaster Med. Public Health Prep..

[23] Lavelle F., Hollywood L., Caraher M., McGowan L., Spence M., Surgenor D., McCloat A., Mooney E., Raats M., Dean M. (2017). Increasing intention to cook from basic ingredients: A randomised controlled study. Appetite.

[24] Mills S.D., Wolfson J.A., Wrieden W.L., Brown H., White M., Adams J. (2020). Perceptions of ‘Home Cooking’: A Qualitative Analysis from the United Kingdom and United States. Nutrients.

[25] Vinkers C.H., van Amelsvoort T., Bisson J.I., Branchi I., Cryan J.F., Domschke K., Howes O.D., Manchia M., Pinto L., de Quervain D. (2020). Stress resilience during the coronavirus pandemic. Eur. Neuropsychopharmacol..

[26] Martin-Neuninger R., Ruby M.B. (2020). What Does Food Retail Research Tell Us About the Implications of Coronavirus (COVID-19) for Grocery Purchasing Habits?. Front. Psychol..

[27] Baker S.R., Farrokhnia A.R., Meyer S., Pagel M., Yannelis C. (2020). How Does Household Spending Respond to an Epidemic? Consumption during the 2020 COVID-19 Pandemic. Rev. Asset Pricing Stud..

[28] Wang E., An N., Gao Z., Kiprop E., Geng X. (2020). Consumer food stockpiling behavior and willingness to pay for food reserves in COVID-19. Food Secur..

[29] Husain W., Ashkanani F. (2020). Does COVID-19 change dietary habits and lifestyle behaviours in Kuwait: A community-based cross-sectional study. Environ. Health Prev. Med..

[30] Laguna L., Fiszman S., Puerta P., Chaya C., Tárrega A. (2020). The impact of COVID-19 lockdown on food priorities. Results from a preliminary study using social media and an online survey with Spanish consumers. Food Qual. Prefer..

[31] Zhang J., Zhao A., Ke Y., Huo S., Ma Y., Zhang Y., Ren Z., Li Z., Liu K. (2020). Dietary Behaviors in the Post-Lockdown Period and Its Effects on Dietary Diversity: The Second Stage of a Nutrition Survey in a Longitudinal Chinese Study in the COVID-19 Era. Nutrients.

[32] Bracale R., Vaccaro C.M. (2020). Changes in food choice following restrictive measures due to Covid-19. Nutr. Metab. Cardiovasc. Dis..

[33] Giacalone D., Frøst M.B., Rodríguez-Pérez C. (2020). Reported Changes in Dietary Habits During the COVID-19 Lockdown in the Danish Population: The Danish COVIDiet Study. Front. Nutr..

[34] Scarmozzino F., Visioli F. (2020). Covid-19 and the Subsequent Lockdown Modified Dietary Habits of Almost Half the Population in an Italian Sample. Foods.

[35] Steele E.M., Rauber F., Costa C.D.S., Leite M.A., Gabe K.T., Louzada M.L.D.C., Levy R.B., Monteiro C.A. (2020). Mudanças alimentares na coorte NutriNet Brasil durante a pandemia de covid-19. Revista Saúde Pública.

[36] Chang H., Meyerhoefer C.D. (2021). COVID-19 and the Demand for Online Food Shopping Services: Empirical Evidence from Taiwan. Am. J. Agric. Econ..

[37] Sidor A., Rzymski P. (2020). Dietary Choices and Habits during COVID-19 Lockdown: Experience from Poland. Nutrients.

[38] Kriaucioniene V., Bagdonaviciene L., Rodríguez-Pérez C., Petkeviciene J. (2020). Associations between Changes in Health Behaviours and Body Weight during the COVID-19 Quarantine in Lithuania: The Lithuanian COVIDiet Study. Nutrients.

[39] Pearl R.L. (2020). Weight Stigma and the “Quarantine-15”. Obesity.

[40] Bhutani S., Cooper J.A. (2020). COVID-19–Related Home Confinement in Adults: Weight Gain Risks and Opportunities. Obesity.

[41] Drywień M., Hamulka J., Zielinska-Pukos M., Jeruszka-Bielak M., Górnicka M. (2020). The COVID-19 Pandemic Lockdowns and Changes in Body Weight among Polish Women. A Cross-Sectional Online Survey PLifeCOVID-19 Study. Sustainability.

[42] Fernandez-Rio J., Cecchini J.A., Mendez-Gimenez A., Carriedo A. (2020). Weight changes during the COVID-19 home confinement. Effects on psychosocial variables. Obes. Res. Clin. Pract..

[43] He M., Xian Y., Lv X., He J., Ren Y. (2020). Changes in Body Weight, Physical Activity, and Lifestyle During the Semi-lockdown Period After the Outbreak of COVID-19 in China: An Online Survey. Disaster Med. Public Health Prep..

[44] Sallis J.F., Adlakha D., Oyeyemi A., Salvo D. (2020). An international physical activity and public health research agenda to inform coronavirus disease-2019 policies and practices. J. Sport Health Sci..

[45] Robinson E., Boyland E., Chisholm A., Harrold J., Maloney N.G., Marty L., Mead B.R., Noonan R., Hardman C.A. (2021). Obesity, eating behavior and physical activity during COVID-19 lockdown: A study of UK adults. Appetite.

[46] Ingram J., Maciejewski G., Hand C.J. (2020). Changes in Diet, Sleep, and Physical Activity Are Associated with Differences in Negative Mood During COVID-19 Lockdown. Front. Psychol..

[47] Schnitzer M., Schöttl S., Kopp M., Barth M. (2020). COVID-19 stay-at-home order in Tyrol, Austria: Sports and exercise behaviour in change?. Public Health.

[48] Błaszczyk-Bębenek E., Jagielski P., Bolesławska I., Jagielska A., Nitsch-Osuch A., Kawalec P. (2020). Nutrition Behaviors in Polish Adults before and during COVID-19 Lockdown. Nutrients.

[49] Yang Y., Koenigstorfer J. (2020). Determinants of physical activity maintenance during the Covid-19 pandemic: A focus on fitness apps. Transl. Behav. Med..

[50] Ruíz-Roso M., Padilha P.D.C., Matilla-Escalante D., Brun P., Ulloa N., Acevedo-Correa D., Peres W.A.F., Martorell M., Carrilho T.R.B., Cardoso L.D.O. (2020). Changes of Physical Activity and Ultra-Processed Food Consumption in Adolescents from Different Countries during Covid-19 Pandemic: An Observational Study. Nutrients.

[51] Ong J.L., Lau T., Massar S.A.A., Chong Z.T., Ng B.K.L., Koek D., Zhao W., Yeo B.T.T., Cheong K., Chee M.W.L. (2021). COVID-19-related mobility reduction: Heterogenous effects on sleep and physical activity rhythms. Sleep.

[52] Castañeda-Babarro A., Arbillaga-Etxarri A., Gutiérrez-Santamaría B., Coca A. (2020). Physical Activity Change during COVID-19 Confinement. Int. J. Environ. Res. Public Health.

[53] Caputo E.L., Reichert F.F. (2020). Studies of Physical Activity and COVID-19 During the Pandemic: A Scoping Review. J. Phys. Act. Health.

[54] López-Bueno R., Calatayud J., Casaña J., Casajús J.A., Smith L., Tully M.A., Andersen L.L., López-Sánchez G.F. (2020). COVID-19 Confinement and Health Risk Behaviors in Spain. Front. Psychol..

