# Supplementary material for: Dietary Habits before and during the COVID-19 Epidemic in Selected European Countries
Source: Nutrients. 2021 May 16;13(5):1690. doi: 10.3390/nu13051690 (PMC8156782; doi:10.3390/nu13051690)
Supplement: Supplementary file 1 [file nutrients-13-01690-s001.zip › nutrients-1173804-supplementary.pdf]

Table S1. Alpha Cronbach test.

α-Cronbach/ split-half reliabilities	p-value
Analyzed variables	
Cardinality-uninterpreted	0
Cardinality-no data	0
Significance level	0.05
Cardinality	1071
Number of items	52
Scale mean	187.047619
Standard deviation of the scale	17.177647
α-Cronabach value of the scale	0.79921
-95% CI for α-Cronabach value of the scale	0.781139
+95% CI for α-Cronabach value of the scale	0.816011
Standard error of measurement	7.697241
Average correlation between item-pairs	0.080723
Standardized α-Cronbach value	0.820344

Table S2. Comparison of adults' dietary habits before and during COVID-19 pandemic n=1071. Wilcoxon test.

Question	p-value	Sum of the positives ranks	Sum of the negatives ranks
Frequency of consumption of 4-5 meals a day	<0.001	61319.5	14535.5
Eating at least one warm meal	<0.001	23694.0	2871.0
Preparing homemade meals	<0.001	104719.5	20030.5
Shopping frequency	<0.001	33744.0	92007.0
Shopping in the supermarket	<0.001	17953.5	92261.5
Shopping in local market-place	0.003	49153.0	74600.0
Shopping online	<0.001	92808.0	2895.0
Ordering readymade meals at restaurants	0.021	57779.5	45505.5
Eat out	<0.001	3187.5	346678.5

Table S3. The frequency of consumption of particular foods before and during COVID-19 for analyzed population n=1071. Wilcoxon test.

Product	p-value	Sum of the positives ranks	Sum of the negatives ranks
Frozen food	<0.001	43936.0	19967.0
Canned food	<0.001	53548.5	14716.5
Sweets and snacks	<0.001	53407.5	21512.5
Diary and eggs	<0.001	14498.5	3646.5
Cereal products	<0.001	14429.0	6481.0
Fats and oil	0.003	8260.0	3216.0
Fruits	0.059	NS	NS
Vegetables and nuts	0.021	31604.0	6622.0
Meat	0.226	NS	NS
Fish and seafood	<0.001	10706.0	23474.0
Coffee	0.006	10647.0	15918.0
Tea	<0.001	16120.0	3186.0
Water	0.000	4428.0	1900.0
Juice and sweets drinks	<0.000001	10082.0	21796.0
Alcohol	0.002188	56476.5	40984.5

(NS – not significant).

Table S4. Nutritional behavior and shopping habits of consumers depending on the country of residence. Kruskal-Wallis analysis of variance (ANOVA).

Question		Poland/United Kingdom	Poland/Austria	Austria/United Kingdom	p-value
Question	1a	NS	NS	NS	0.136*
	1b	NS	0.030	NS	0.033
Question	2a	<0.001	<0.001	NS	<0.001
	2b	<0.001	NS	NS	<0.001
Question	3a	<0.001	<0.001	NS	<0.001
	3b	<0.001	<0.001	NS	<0.001
Question	4a	<0.001	0.004	NS	<0.001
	4b	NS	NS	NS	0.256*
Question	5a	<0.001	<0.001	NS	<0.001
	5b	<0.001	<0.001	NS	<0.001
Question	6a	0.002	0.018	NS	0.001
	6b	NS	NS	NS	0.118*
Question	7a	NS	NS	NS	0.082*
	7b	0.029	NS	NS	0.032
Question	8a	<0.001	<0.001	NS	<0.001
	8b	<0.001	<0.001	NS	<0.001
Question	9a	<0.001	<0.001	NS	<0.001
	9b	0.002	<0.001	NS	0.006

*p>0.05, NS – not significant, a-before pandemic; b-during pandemic; 1a,b- frequency of consumption of 4-5 meals a day; 2a,b -at least one warm meal; 3a,b- frequency of preparation of home-made meals; 4a,b- shopping frequency; 5a,b- frequency of shopping in the supermarket; 6a,b- shopping from the local suppliers; 7a,b- frequency of shopping online; 8a,b- ordering ready-made meals at restaurants; 9a,b- frequency of eating out.

Table S5. Nutritional behavior of consumers depending on the country of residence. Kruskal-Wallis analysis of variance (ANOVA)

Question		Poland/United Kingdom	Poland/Austria	Austria/United Kingdom	p-value
Question	10a	0.001	NS	NS	0.002
	10b	<0.001	0.006	NS	<0.001
Question	11a	0.031	NS	NS	0.033
	11b	0.004	NS	0.019	0.003
Question	12a	<0.001	NS	0.009	<0.001
	12b	NS	NS	NS	0.745*
Question	13a	NS	NS	NS	0.613
	13b	<0.001	NS	NS	<0.001
Question	14a	<0.001	<0.001	NS	<0.001
	14b	<0.001	<0.0001	NS	<0.001
Question	15a	NS	NS	NS	0.071*
	15b	NS	NS	NS	0.082*
Question	16a	0.001	<0.0001	NS	<0.001
	16b	0.029	NS	NS	0.033
Question	17a	<0.001	<0.001	NS	<0.001
	17b	<0.001	<0.001	NS	<0.001
Question	18a	<0.001	<0.001	NS	<0.001
	18b	0.002	0.001	NS	<0.001
Question	19a	NS	0.001	0.010	<0.001
	19b	NS	NS	NS	0.095*

Question	20a	<0.001	<0.001	0.018	<0.001
	20b	<0.001	<0.001	<0.001	<0.001
Question	21a	0.014	NS	<0.001	<0.001
	21b	0.001	NS	<0.001	<0.001
Question	22a	NS	NS	NS	0.088*
	22b	NS	NS	NS	0.063*
Question	23a	<0.001	<0.001	NS	0.001
	23b	0.006	NS	NS	0.006
Question	24a	NS	NS	NS	0.448*
	24b	NS	NS	NS	0.260*

*p>0.05, NS – not significant, a-before pandemic; b-during pandemic; frequency of consumption-10a,b- frozen food; 11a,b -canned food; 12a,b- sweets and snacks; 13a,b- dairy and eggs; 14a,b- cereal products; 15a,b- fats, oils; 16a,b- fruits; 17a,b- vegetables and nuts; 18a,b- meat; 19a,b- fish and seafood; 20a,b-coffe; 21a,b-tea; 22a,b-water; 23a,b-juice and sweets drink; 24a,b-alcohol.

Questionnaire S1. Comparison of dietetary behaviours before and during the COVID19 pandemic.

Dear Sir/Madam,

The Sars-COV-2 pandemic has significant impact on our lives, including day-to-day activities and habits. The aim of this study is to examine the dietetary behaviours before and during the pandemic among the society.

We would like you to complete the provided 10-minute questionnaire. It focuses on the dietetary behaviour before and during the pandemic.

Each question requires 2 answers – first is about the frequency of specific action before the pandemic and the second is about the frequency of the action during pandemic. Please answer every question. If you are uncertain about how to answer a question then do the best you can, but please do not leave a question blank.

We really appreciate you taking this survey.

Yours sincerely,

Department of Food Commodity Science

Medical University in Gdansk

1a) Before the pandemic I used to eat 4-5 meals per day

□ Never or almost never

□ Once or less per month

□ Few times per month

□ Few times per week

□ Once a day

1b) During the pandemic I eat 4-5 meals per day

□ Never or almost never

□ Once or less per month

□ Few times per month

□ Few times per week

□ Once a day

2a) Before the pandemic I used to go shopping for food

□ Never or almost never

□ Once or less per month

□ Few times per month

2b) During the pandemic I shop for food

□ Never or almost never

□ Once or less per month

□ Few times per month

□ Few times per week

□ Once a day

□ Few times per day

□ Few times per week

□ Once a day

□ Few times per day

3a) Before the pandemic I used to do shopping in the supermarket

□ Never or almost never

□ Once or less per month

□ Few times per month

□ Few times per week

□ Once a day

□ Few times per day

3b) During the pandemic I shop in the supermarket

□ Never or almost never

□ Once or less per month

□ Few times per month

□ Few times per week

□ Once a day

□ Few times per day

4a) Before the pandemic I used to do shopping in the convenience store or local marketplace

□ Never or almost never

□ Once or less per month

□ Few times per month

□ Few times per week

□ Once a day

□ Few times per day

4b) During the pandemic I shop in the convenience store or local marketplace

□ Never or almost never

□ Once or less per month

□ Few times per month

□ Few times per week

□ Once a day

□ Few times per day

5a) Before the pandemic I used to do shopping online

□ Never or almost never

□ Once or less per month

□ Few times per month

□ Few times per week

□ Once a day

□ Few times per day

5b) During the pandemic I do shopping online

□ Never or almost never

□ Once or less per month

□ Few times per month

□ Few times per week

□ Once a day

□ Few times per day

6a) Before the pandemic I used to cook at home

□ Never or almost never

□ Once or less per month

□ Few times per month

□ Few times per week

□ Once a day

□ Few times per day

6b) During the pandemic I cook at home

□ Never or almost never

□ Once or less per month

□ Few times per month

□ Few times per week

□ Once a day

□ Few times per day

7a) Before the pandemic I used to order food from restaurants or caterers

□ Never or almost never

7b) During the pandemic I order food from restaurants or caterers

□ Never or almost never

□ Once or less per month
□ Few times per month
□ Few times per week
□ Once a day
□ Few times per day

□ Once or less per month
□ Few times per month
□ Few times per week
□ Once a day
□ Few times per day

8a) Before the pandemic I used to eat out

□ Never or almost never
□ Once or less per month
□ Few times per month
□ Few times per week
□ Once a day
□ Few times per day

8b) During the pandemic I eat out

□ Never or almost never
□ Once or less per month
□ Few times per month
□ Few times per week
□ Once a day
□ Few times per day

9a) Before the pandemic I used to eat at least 1 warm meal

□ Never or almost never
□ Once or less per month
□ Few times per month
□ Few times per week
□ Once a day
□ Few times per day

9b) During the pandemic I eat at least 1 warm meal

□ Never or almost never
□ Once or less per month
□ Few times per month
□ Few times per week
□ Once a day
□ Few times per day

10a) Before the pandemic I used to buy frozen food

□ Never or almost never
□ Once or less per month
□ Few times per month
□ Few times per week
□ Once a day
□ Few times per day

10b) During the pandemic I buy frozen food

□ Never or almost never
□ Once or less per month
□ Few times per month
□ Few times per week
□ Once a day
□ Few times per day

11a) Before the pandemic I used to buy long shelf life products (cans, jars, concentrates)

□ Never or almost never
□ Once or less per month
□ Few times per month
□ Few times per week
□ Once a day
□ Few times per day

11b) During the pandemic I buy long shelf life products (cans, jars, concentrates)

□ Never or almost never
□ Once or less per month
□ Few times per month
□ Few times per week
□ Once a day
□ Few times per day

12a) Before the pandemic I used to consume snacks and sweets

□ Never or almost never

□ Once or less per month

□ Few times per month

□ Few times per week

□ Once a day

□ Few times per day

12b) During the pandemic I consume snacks and sweets

□ Never or almost never

□ Once or less per month

□ Few times per month

□ Few times per week

□ Once a day

□ Few times per day

13a) Before the pandemic I used to consume dairy and eggs

□ Never or almost never

□ Once or less per month

□ Few times per month

□ Few times per week

□ Once a day

□ Few times per day

13b) During the pandemic I consume dairy and eggs

□ Never or almost never

□ Once or less per month

□ Few times per month

□ Few times per week

□ Once a day

□ Few times per day

14a) Before the pandemic I used to consume cereal products

□ Never or almost never

□ Once or less per month

□ Few times per month

□ Few times per week

□ Once a day

□ Few times per day

14b) During the pandemic I consume cereal products

□ Never or almost never

□ Once or less per month

□ Few times per month

□ Few times per week

□ Once a day

□ Few times per day

15a) Before the pandemic I used to consume fats

(eg. butter,margarine, olive oil, rapeseed oil etc.)

□ Never or almost never

□ Once or less per month

□ Few times per month

□ Few times per week

□ Once a day

□ Few times per day

15b) During the pandemic I consume fats

(eg. butter,margarine, olive oil, rapeseed oil etc.)

□ Never or almost never

□ Once or less per month

□ Few times per month

□ Few times per week

□ Once a day

□ Few times per day

16a) Before the pandemic I used to consume fruits

□ Never or almost never

□ Once or less per month

□ Few times per month

□ Few times per week

□ Once a day

16b) During the pandemic I consume fruits

□ Never or almost never

□ Once or less per month

□ Few times per month

□ Few times per week

□ Once a day

□ Few times per day

□ Few times per day

17a) Before the pandemic I used to consume vegetables and nuts

□ Never or almost never
□ Once or less per month
□ Few times per month
□ Few times per week
□ Once a day
□ Few times per day

17b) During the pandemic I consume vegetables and nuts

□ Never or almost never
□ Once or less per month
□ Few times per month
□ Few times per week
□ Once a day
□ Few times per day

18a) Before the pandemic I used to consume meat and meat products

□ Never or almost never
□ Once or less per month
□ Few times per month
□ Few times per week
□ Once a day
□ Few times per day

18b) Before the pandemic I used to consume meat and meat products

□ Never or almost never
□ Once or less per month
□ Few times per month
□ Few times per week
□ Once a day
□ Few times per day

19a) Before the pandemic I used to consume fish and seafood

□ Never or almost never
□ Once or less per month
□ Few times per month
□ Few times per week
□ Once a day
□ Few times per day

19b) During the pandemic I consume fish and seafood

□ Never or almost never
□ Once or less per month
□ Few times per month
□ Few times per week
□ Once a day
□ Few times per day

20a) Before the pandemic I used to drink coffee

□ Never or almost never
□ Once or less per month
□ Few times per month
□ Few times per week
□ Once a day
□ Few times per day

20b) During the pandemic I drink coffee

□ Never or almost never
□ Once or less per month
□ Few times per month
□ Few times per week
□ Once a day
□ Few times per day

21a) Before the pandemic I used to drink tea

□ Never or almost never
□ Once or less per month
□ Few times per month

21b) Before the pandemic I drink tea

□ Never or almost never
□ Once or less per month
□ Few times per month

□ Few times per week
□ Once a day
□ Few times per day

□ Few times per week
□ Once a day
□ Few times per day

22a) Before the pandemic I used to drink water
□ Never or almost never
□ Once or less per month
□ Few times per month
□ Few times per week
□ Once a day
□ Few times per day

22b) During the pandemic I drink water
□ Never or almost never
□ Once or less per month
□ Few times per month
□ Few times per week
□ Once a day
□ Few times per day

23a) Before the pandemic I used to drink juice and sweet drinks
□ Never or almost never
□ Once or less per month
□ Few times per month
□ Few times per week
□ Once a day
□ Few times per day

23b) During the pandemic I drink juice and sweet drinks
□ Never or almost never
□ Once or less per month
□ Few times per month
□ Few times per week
□ Once a day
□ Few times per day

24a) Before the pandemic I used to drink alcohol
□ Never or almost never
□ Once or less per month
□ Few times per month
□ Few times per week
□ Once a day
□ Few times per day

24b) During the pandemic I drink alcohol
□ Never or almost never
□ Once or less per month
□ Few times per month
□ Few times per week
□ Once a day
□ Few times per day

26a) Before the pandemic I used exercise
□ Never or almost never
□ Once or less per month
□ Few times per month
□ Few times per week
□ Once a day
□ Few times per day

26b) During the pandemic I exercise
□ Never or almost never
□ Once or less per month
□ Few times per month
□ Few times per week
□ Once a day
□ Few times per day

GENDER	AGE	EDUCATION	DURING PANDEMIC
□ Woman<br>□ Man<br>□ Other		□ Primary education<br>□ Secondary education<br>□ Higher education	□ I've gained weight<br>□ I've lost weight<br>□ My body mass haven't changed
